# Supplementary material for: Different Methods to Modify the Hydrophilicity of Titanium Implants with Biomimetic Surface Topography to Induce Variable Responses in Bone Marrow Stromal Cells
Source: Biomimetics (Basel). 2024 Apr 10;9(4):227. doi: 10.3390/biomimetics9040227 (PMC11048143; doi:10.3390/biomimetics9040227)
Supplement: Supplementary file 1 [file biomimetics-09-00227-s001.zip › biomimetics-2831481-supplementary.pdf]

Supplementary Figures

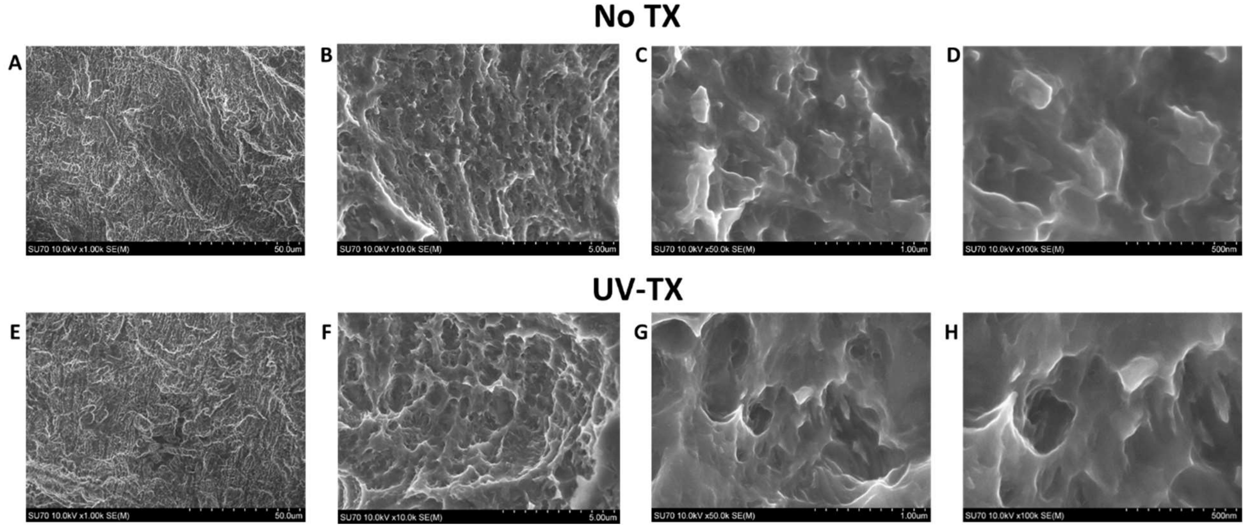

Supp. Figure S1: Scanning electron micrographs at 1kX (A,E), 10 kX (B,F), 50 kX (C,G) and 100 kX (D,H) magnification of untreated Ti6Al4V surfaces (top row) and Ti6Al4V surfaces cleaned by UV treatment (bottom row).

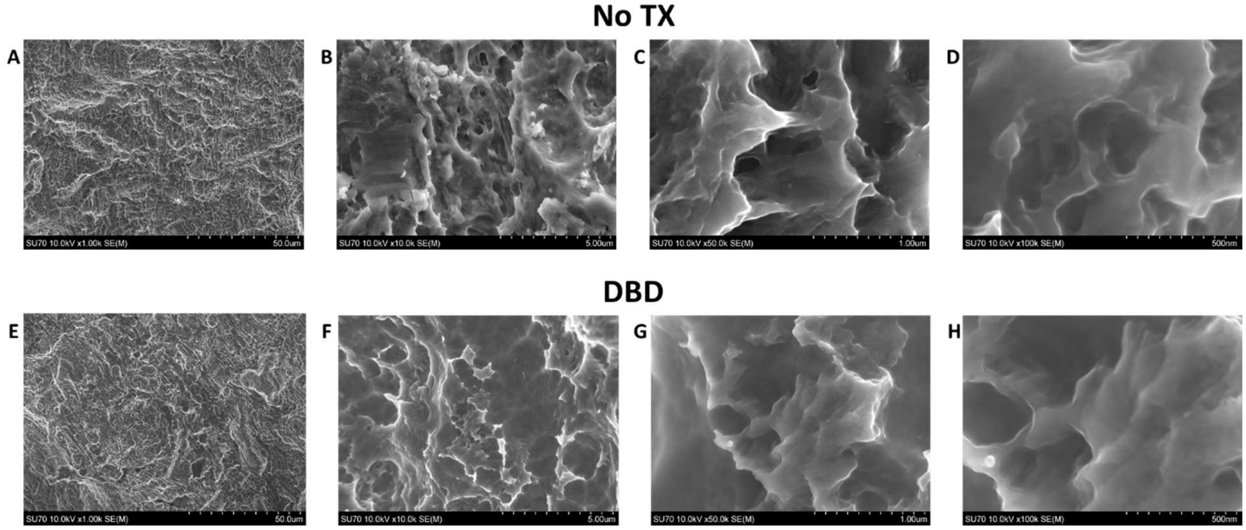

Supp. Figure S2: Scanning electron micrographs at 1kX (A,E), 10 kX (B,F), 50 kX (C,G) and 100 kX (D,H) magnification of untreated Ti6Al4V surfaces (top row) and Ti6Al4V surfaces treated with DBD plasma (bottom row).

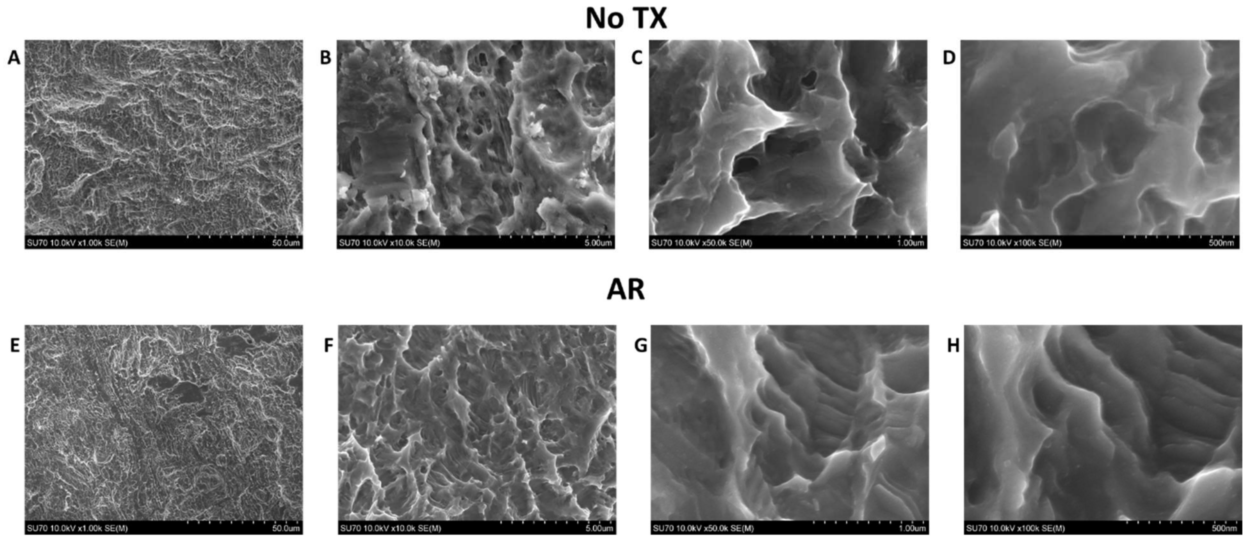

Supp. Figure S3: Scanning electron micrographs at 1kX (A,E), 10 kX (B,F), 50 kX (C,G) and 100 kX (D,H) magnification of untreated Ti6Al4V surfaces (top row) and Ti6Al4Vtreated with argon gas plasma (bottom row).

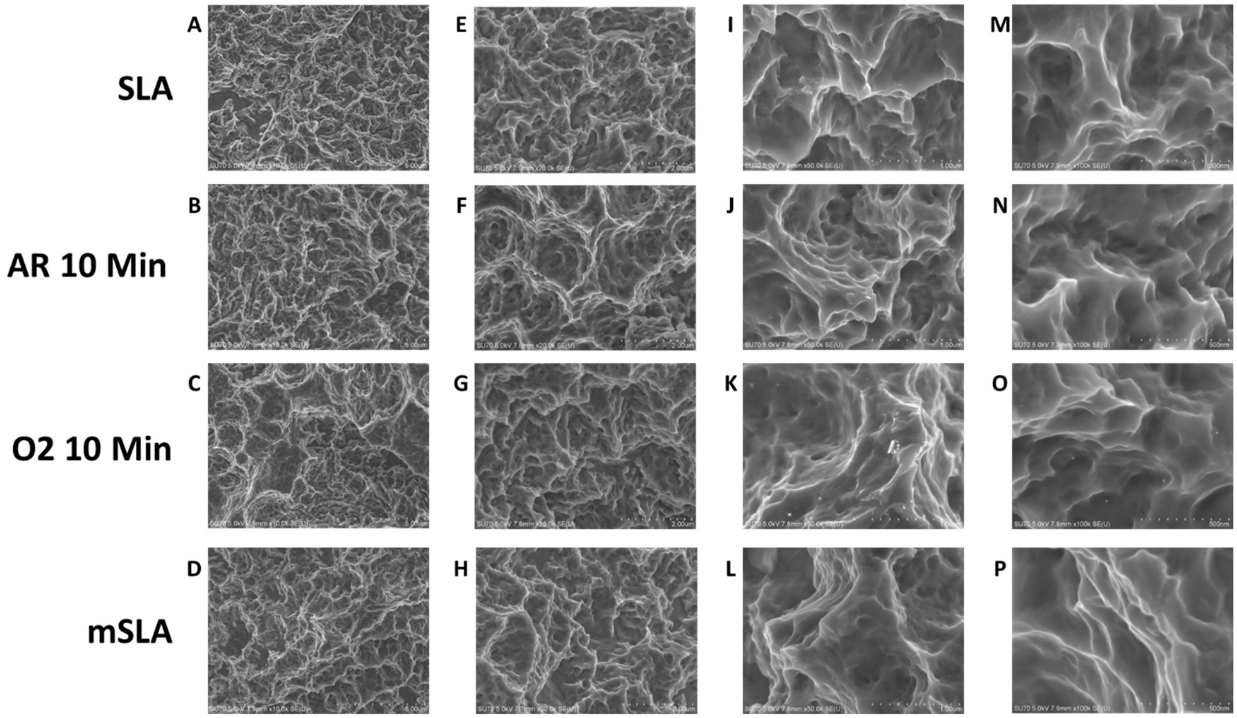

Supp. Figure S4: Scanning electron micrographs at 1kX (A,E,I,M), 10 kX (B,F,J,N), 50 kX (C,G,K,O) and 100 kX (D,H,L,P) magnification of untreated SLA surfaces, SLA surfaces cleaned with argon plasma cleaner for 10 minutes, SLA surfaces cleaned with oxygen plasma cleaner for 10 minutes, and untreated modSLA.

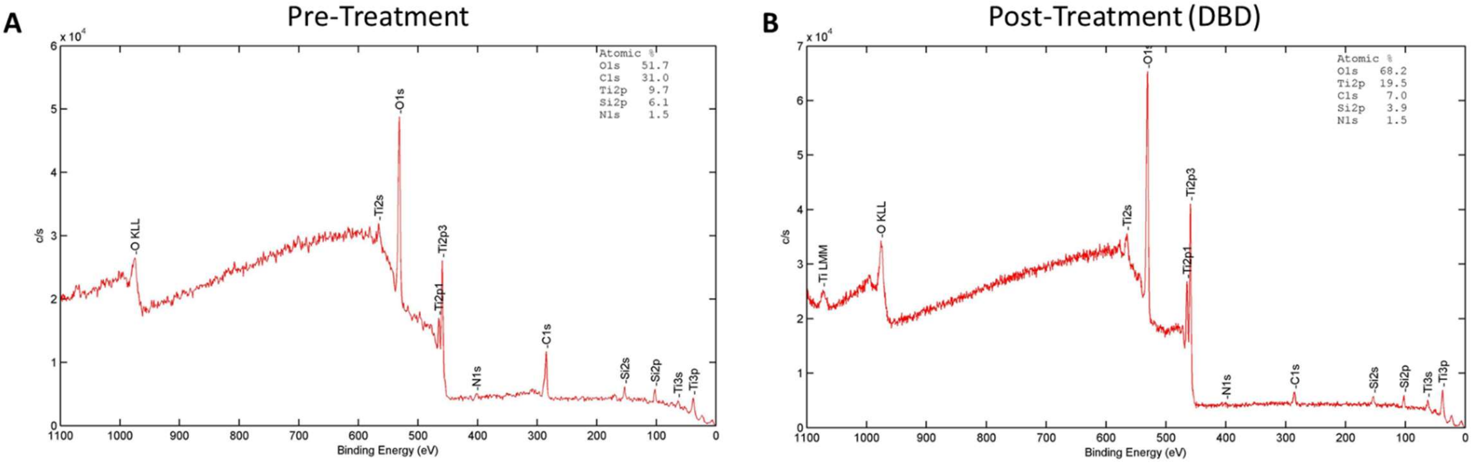

Supp. Figure S5: Representative XPS spectra of survey scans before (A) and after plasma treatment (B) A scan following DBD plasma treatment is shown to display changes in elemental composition following treatment.

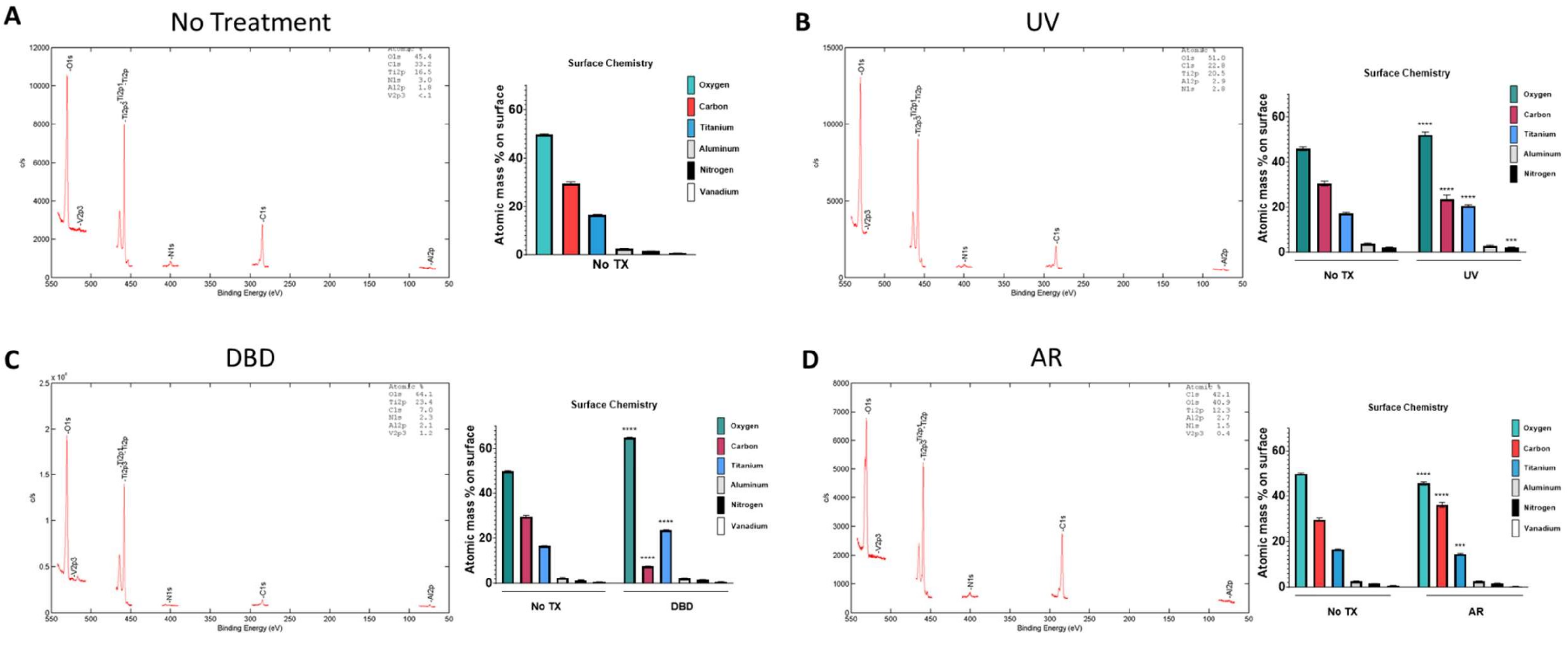

Supp. Figure S6: Representative XPS spectra of region scans for elements of interest for No Treatment (A), UV treatment (B), DBD plasma treatment (C), and Argon plasma treatment (D).
